# Slow growth as a strategy for plant longevity

**DOI:** 10.1038/s41467-026-76765-0

**Published:** 2026-08-13

**Authors:** Jan Binter, Michael Peter Nobis, Jan Altman, Ulf Büntgen, Alan Crivellaro, Pierre Liancourt, Jiří Doležal

**Affiliations:** 1 Institute of Botany of the Czech Academy of Sciences, Průhonice, Czech Republic; 2 Faculty of Science, University of South Bohemia, České Budějovice, Czech Republic; 3 Swiss Federal Research Institute WSL, Birmensdorf, Switzerland; 4 Faculty of Forestry and Wood Sciences, Czech University of Life Sciences, Prague, Czech Republic; 5 Department of Geography, University of Cambridge, Cambridge, UK; 6 Global Change Research Institute (CzechGlobe), Czech Academy of Sciences, Brno, Czech Republic; 7 Department of Geography, Faculty of Science, Masaryk University, Brno, Czech Republic; 8 Department of Agricultural, Forest and Food Sciences, Università degli Studi di Torino, Grugliasco, Torino Italy; 9 Forest Biometrics Laboratory, Faculty of Forestry, “Stefan cel Mare” University of Suceava, Suceava, Romania; 10 Department of Botany, State Museum of Natural History Stuttgart, Stuttgart, Germany; 11 Plant Ecology Group, University of Tübingen, Tübingen, Germany

**Keywords:** Macroecology, Ecosystem ecology

## Abstract

Ecological theories predict a fundamental trade-off between fast growth and the probability of achieving longevity, with far-reaching consequences for ecosystem resilience and carbon residence time. Tree-ring studies have revealed a globally consistent negative relationship between growth rate and lifespan, yet comparable large-scale evidence for such trade-offs beyond trees remains scarce, largely due to methodological limitations. Here, we exploit annual growth rings formed by secondary thickening in perennial herbaceous plants to test whether the growth–longevity trade-off represents a widespread axis of plant strategies extending across life forms. Using an extensive dataset of over 3100 herbaceous and small woody species, including lianas, sampled across five continents and ecosystems ranging from the Arctic to hot semideserts, we reveal a robust association between slow growth and longevity across life forms and environmental conditions. Moreover, the growth rate exhibited a strong temperature dependence, giving rise to an apparent longevity–temperature relationship, highlighting ecological relevance of growth–longevity coupling. Together, our findings identify the growth–longevity trade-off as a widespread constraint on plant strategies, with important implications for predicting vegetation dynamics, ecosystem stability, and the persistence of terrestrial carbon storage in a changing climate.

## Abstract (web summary)

Plant longevity shapes ecosystem functioning and carbon storage, but evidence for its relationship with growth beyond trees has been limited. The authors show that slow growth consistently predicts greater longevity across diverse plant life forms and climatic conditions.

## Introduction

Longevity is a fundamental trait in plant ecology, with profound implications for population stability, resilience to climate change, and terrestrial carbon sequestration^1–4^. Despite its theoretical and practical importance, the drivers of the striking variation in plant longevity remain incompletely understood. Both theoretical frameworks and empirical evidence suggest that the capacity to attain a long lifespan is linked to reduced growth rate^5–7^, implying a trade-off between investment in rapid growth and long-term persistence^8,9^. As global warming alters plant growth dynamics^10,11^, clarifying this relationship may be critical for predicting climate-driven shifts in plant longevity, with cascading effects on ecosystem resilience and carbon pools^5^. Although the growth–longevity relationship has been documented at the global scale for trees^7,12–14^, comparable large-scale evidence across other life forms remains scarce^9^.

Major ecological frameworks, such as r–K selection theory, Grime’s CSR triangle, and the fast–slow plant economics spectrum, predict that stress-tolerant species tend to exhibit long lifespans, while ruderal species prioritize rapid reproductive turnover at the expense of persistence^15–19^. Similarly, plants that maximize early growth to gain a competitive advantage in light and nutrient acquisition, often coupled with high reproductive investment at young ages, typically allocate fewer resources to tolerate stress and consequently rarely achieve long lifespans^6,20,21^. In contrast, a reduced growth rate is associated with greater investment in defense against insects and pathogens^22^, increased production of metabolically costly compounds conferring resistance to abiotic stress^23^, and improved biomechanical properties of tissues^24^, enhancing long-term survival.

**Fig. 1:**
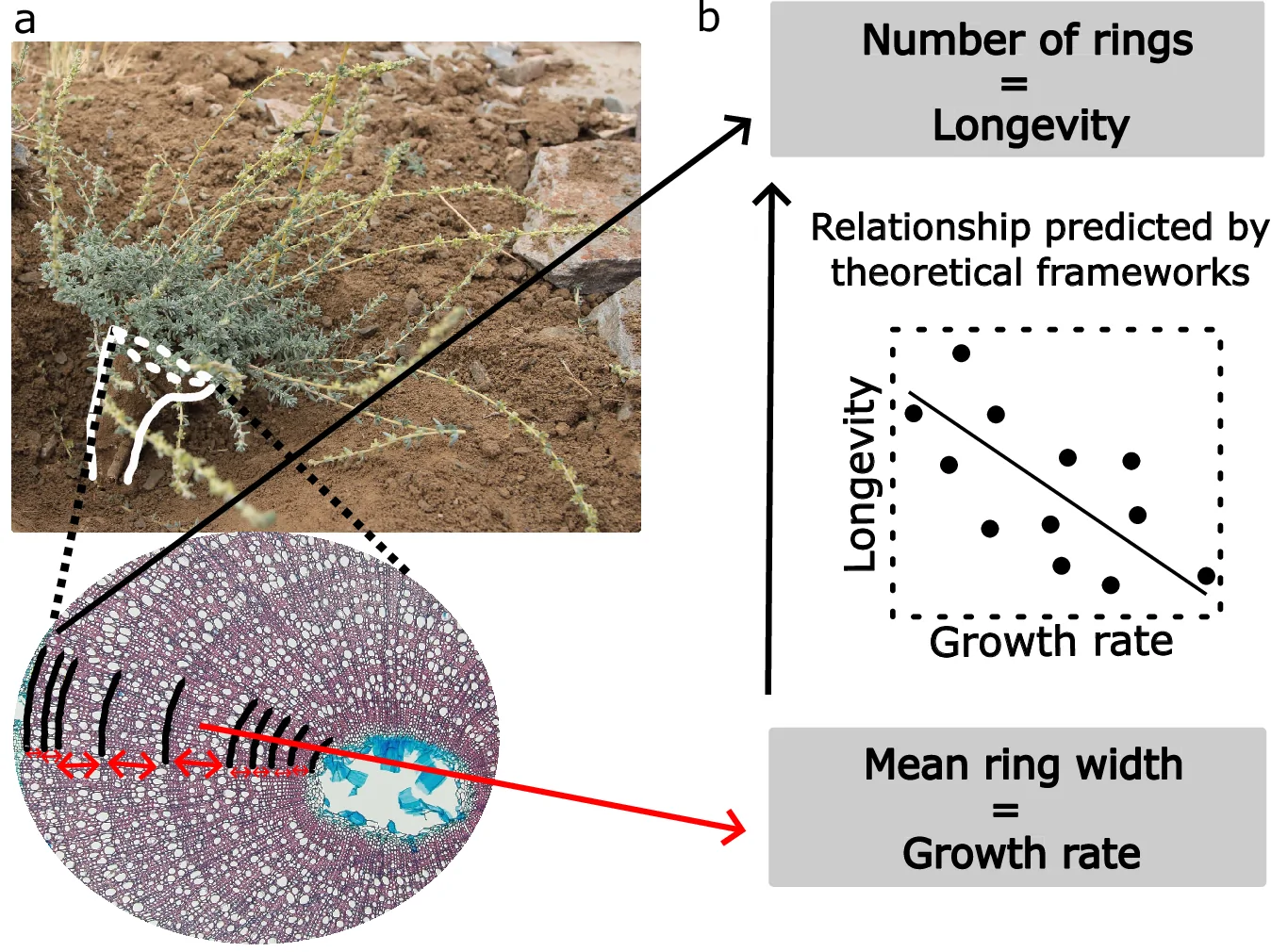
Conceptual framework for analyzing the growth–longevity trade-off in herbaceous plants. **a** Anatomical cross-section of the root crown of a perennial herb showing annual growth rings formed by secondary thickening. Individual rings are delimited by black lines, and their width is indicated by red arrows. **b** The growth–longevity trade-off predicted by major ecological frameworks, which in perennial herbs can be tested as a relationship between mean ring width (growth rate) and the maximum number of rings observed for a mature individual within a species (longevity).

Recent large-scale tree-ring analyses have provided robust evidence for a globally consistent association between slow growth and a higher probability of achieving longevity in trees^7,12,14^. Because tree rings record annual growth rates and individual age simultaneously, trees constitute a methodologically powerful organism for testing fast–slow life-history concepts. Similar trade-offs have also been documented in herbaceous species, primarily within specific taxa or environmental contexts^6,25,26^. Although larger-scale studies on life forms other than trees exist^9,27^, they are limited by the number of species included and often focus on demographic metrics such as survivorship curves, which partly constrain their comparability to evidence derived from tree-ring analyses. Thus, extrapolating these tree-centric and context-specific patterns to the full diversity of vascular plants remains challenging, leaving unresolved whether the growth–longevity association represents a general and potentially widespread constraint shaping plant life histories. Recent advances in anatomical methods now enable annual growth increments in secondary-thickening stems and roots of herbaceous plants to be quantified in a manner analogous to tree-ring analysis^6,25^ (Fig. 1). This methodological breakthrough enables direct empirical testing of a fundamental theoretical question: Does the inverse relationship between growth and longevity extend across diverse plant life forms and ecosystems?

Addressing this question is increasingly urgent, as mounting evidence indicates that climate change is altering plant growth rates across a wide range of life forms^10,26,28^. Warming may enhance growth in some contexts^26,29^, while intensified environmental stressors can constrain growth in others^30,31^. Under the growth–longevity trade-off, such climate-driven shifts in growth rates are expected to translate into changes in lifespan: altered environmental favorability can redirect resource allocation between growth and stress tolerance, thereby modifying long-term persistence^6,18^. These processes may also reshape community composition by changing the relative abundance of long-lived species. Consequently, quantifying the relationship between growth and longevity across plant life forms and ecosystems may inform predictions of vegetation responses to future climates, given the central role of longevity in vegetation resilience to climate change^1^. Beyond community dynamics, plant longevity has important implications for terrestrial carbon cycling^32–34^, as it determines how long carbon remains sequestered in biomass before being released through mortality and decomposition^4^. Growth-mediated changes in plant longevity may therefore represent a critical, yet underappreciated, control on future carbon storage.

Here, we analyze annual growth rings from more than 3100 herbaceous and small-woody plant species, including hemicryptophytes, chamaephytes, nanophanerophytes, and lianas, sampled across five continents and spanning ecosystems from Arctic and alpine environments to temperate lowlands and hot semideserts. We define species longevity as the maximum observed age of a living individual within a species, following previous studies across diverse plant life forms^6,12–14,25^. Using this large, cross-ecosystem dataset, we test a central but still unresolved hypothesis underlying major ecological theories: that the probability of achieving greater longevity is intrinsically linked to reduced growth rates across plant life forms and environmental contexts. We additionally examine how annual growth, longevity, and temperature interact to assess whether the growth–longevity trade-off mediates climatic effects on lifespan. Specifically, we hypothesize that warmer conditions promote faster plant growth, which in turn contributes to reduced longevity. Our results support these hypotheses and demonstrate that the growth–longevity trade-off is widespread across diverse plant life forms and ecosystems, while also contributing to broad climatic patterns in plant longevity.

## Results

### Growth–longevity relationship across life forms

**Fig. 2:**
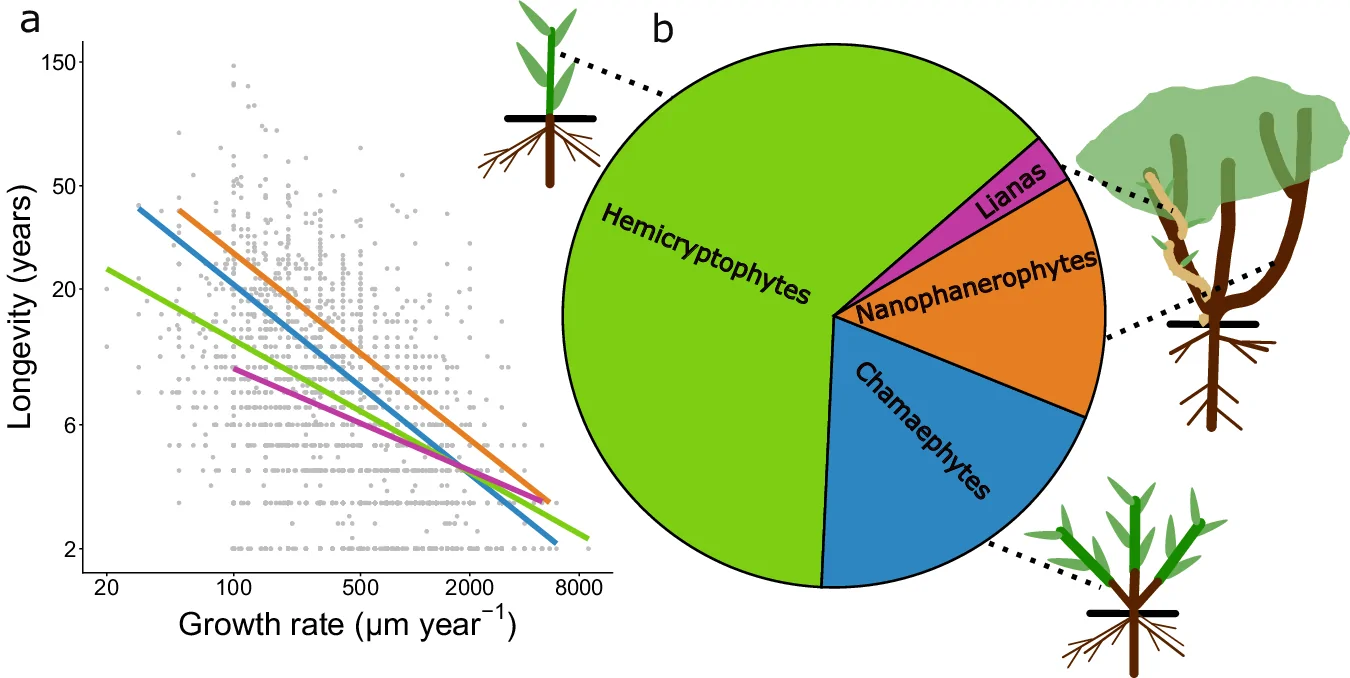
Growth–longevity relationship across plant life forms. **a** Modeled relationship between growth and longevity for each plant life form. All relationships are significant at α = 0.05 and were estimated using phylogenetic models; both axes are shown on a logarithmic scale. Colors correspond to plant life forms. **b** Schematic representation of the studied plant life forms, with perennial organs highlighted in brown. The pie chart shows the proportional representation of each life form in the dataset.

Our analysis revealed a consistent negative association between growth rates and longevity across studied life forms and ecosystems (*R*² = 0.22; *P* < 0.0001). However, the interaction between growth rate and life form was significant (*P* < 0.0001), indicating that the steepness of the growth–longevity relationship differs among life forms (Fig. 2a and Supplementary Table 1). Pairwise comparisons showed significant differences in slopes between hemicryptophytes and chamaephytes (*P* < 0.0001), hemicryptophytes and nanophanerophytes (*P* < 0.001), chamaephytes and lianas (*P* < 0.01), and nanophanerophytes and lianas (*P* = 0.01).

Despite these differences, the relationship was significant within all life forms. When analyzed separately, nanophanerophytes (*R*^2^ = 0.37; *P* < 0.0001) and chamaephytes (*R*^2^ = 0.34; *P* < 0.0001), both characterized by perennial, self-supporting above-ground structures (Fig. 2b), exhibited the strongest negative association between growth rate and longevity. Hemicryptophytes, whose perennial organs remain below ground and are partly buffered from adverse conditions, showed a moderate negative association (*R*^2^ = 0.23; *P* < 0.0001). In contrast, lianas, which rely on external mechanical support, displayed the weakest association (*R*^2^ = 0.14; *P* < 0.001).

Both longevity and growth rates varied markedly among plant life forms. Nanophanerophytes exhibited the greatest mean longevity (average 12.9 years; range 2–124 years), and an average growth rate of 1012 µm year^−1^ (range 50–5500 µm yr^−1^). Chamaephytes showed a comparable mean longevity (12.7 years; range 2–145 years) but substantially lower mean growth rate (559.1 µm yr^−1^; range 30–6000 µm yr^−1^). Hemicryptophytes were shorter-lived, with a mean longevity of 6.4 years (range 2–101 years), and a mean growth rate of 592 µm yr^−1^ (range 20–9000 µm yr^−1^). Lianas exhibited the lowest mean longevity (6.2 years; range 2–37 years) and a mean growth rate of 749.5 µm yr^−1^ (range 100–5000 µm yr^−1^). The maximum longevity recorded in our dataset was 145 years for *Dryas integrifolia* from the Arctic tundra, which exhibited an average annual growth rate of 100 µm yr^−1^. In contrast, the fastest-growing species, *Malva moschata* from temperate lowlands, grew at 9000 µm yr^−1^ and had a longevity of only two years. The slowest growth rate (20 µm year^−1^) was observed in *Aphragmus oxycarpus* from alpine regions, which reached longevity of 12 years.

### Growth–longevity relationship across ecosystems

**Fig. 3:**
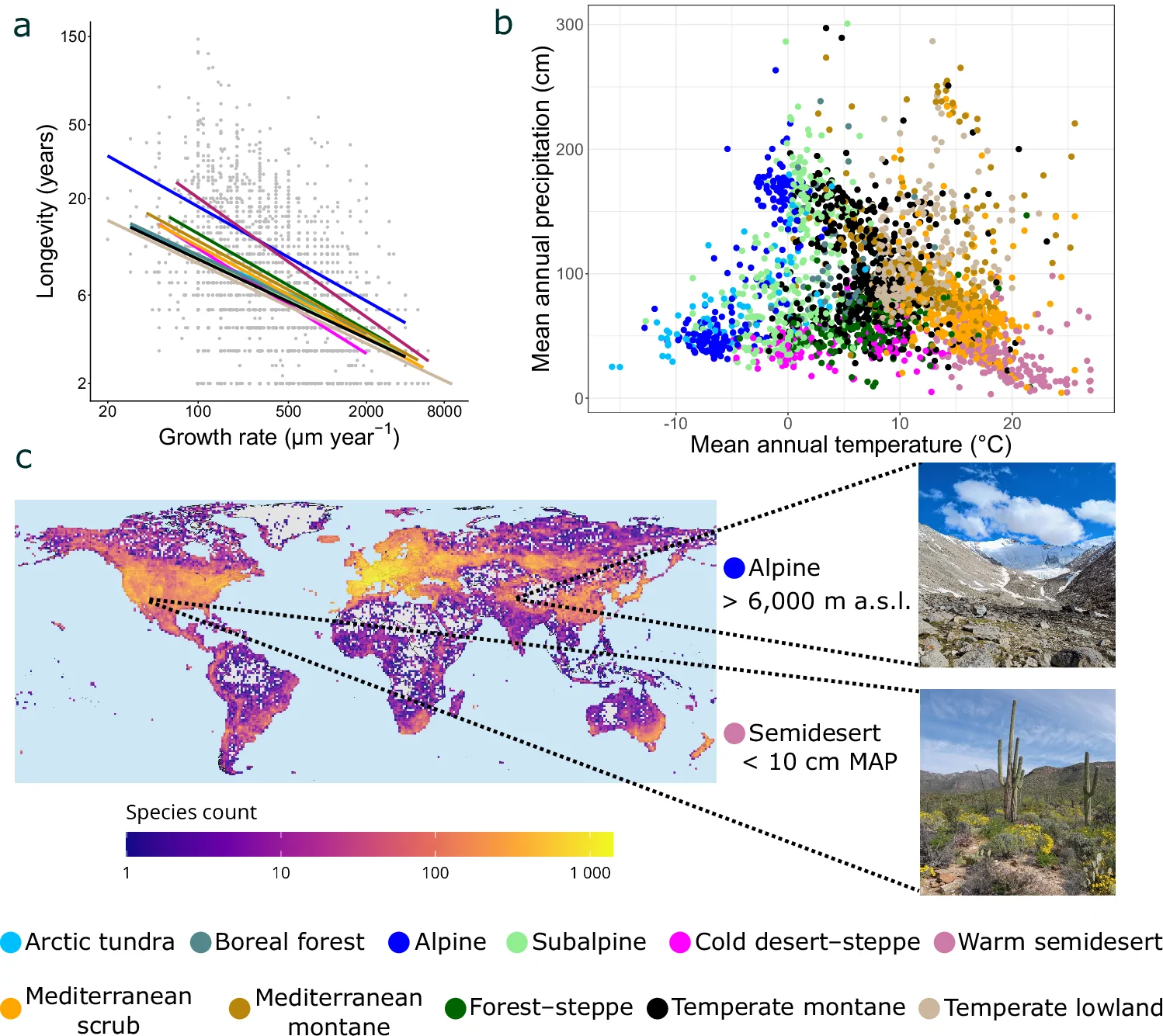
Growth–longevity relationship across contrasting ecosystems. **a** Modeled relationship between growth and longevity for each ecosystem. All relationships are significant at α = 0.05 and were estimated using phylogenetic models; both axes are shown on a logarithmic scale. Colors denote ecosystem categories. **b** Species’ climatic preferences expressed as mean annual precipitation (MAP) versus mean annual temperature, illustrating the range of climatic conditions represented in the study. **c** Global species counts derived from GBIF (Global Biodiversity Information Facility) occurrence data were used to estimate species’ climate preferences. Counts were obtained by combining presence rasters across taxa at 1.0° resolution, while climate preferences were calculated at 30 arc-second resolution. Photographs illustrate some of the most climatically challenging ecosystems included in the study.

The interaction between growth rate and ecosystem was not significant (*P* = 0.40), indicating that the slope of the relationship between growth and longevity is consistent across environmental conditions (Supplementary Table 1 and Fig. 3). However, the proportion of explained variability differed among ecosystems (Supplementary Table 1), with plants from warm semideserts showing by far the strongest association between growth and longevity (*R*² = 0.43).

### Temperature dependence of growth and longevity

**Fig. 4:**
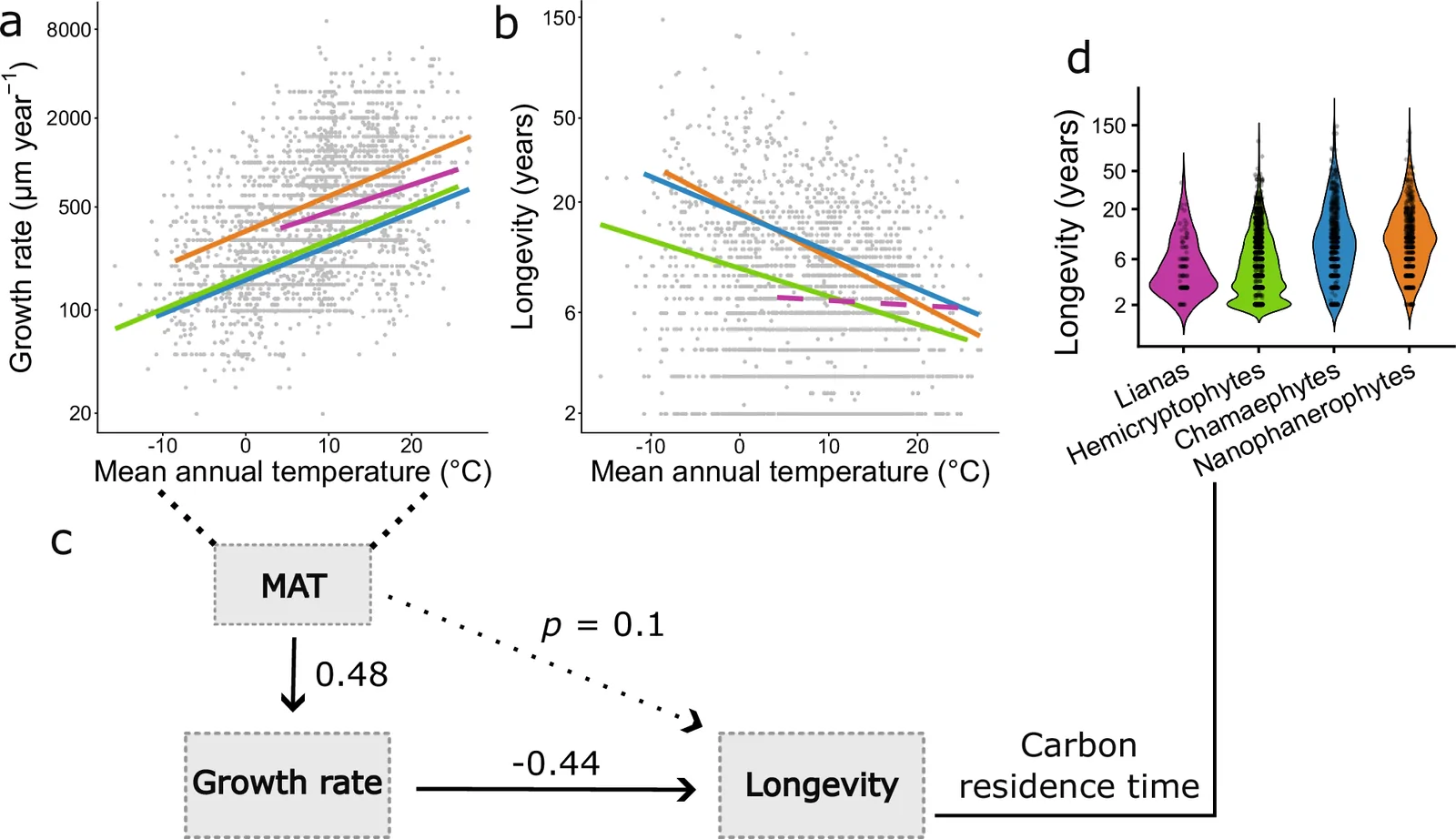
Growth and longevity in relation to mean annual temperature. **a** Modeled relationship between growth rate and mean annual temperature (MAT) for each life form. All relationships are significant at α = 0.05. Colors correspond to plant life forms. **b** Modeled relationship between longevity and mean annual temperature (MAT) for each life form. Solid lines indicate relationships significant at α = 0.05. All relationships in **a** and **b** were estimated using phylogenetic models; the y axes are logarithmic. **c** A path diagram illustrating the associations among MAT, growth, and longevity. Solid lines indicate statistically significant paths (α = 0.05, standardized coefficients shown), whereas the dashed line represents a non-significant path. The effect of MAT on longevity is largely indirect, mediated through growth. **d** Longevity of each life form, highlighting the associated potential for long-term carbon sequestration.

Growth rate was positively associated with mean annual temperature (MAT; *R*^2^ = 0.16, *P* < 0.0001; Fig. 4a), whereas longevity showed a weaker but significant negative relationship with MAT (*R*^2^ = 0.04, *P* < 0.0001; Fig. 4b). However, structural equation modeling (SEM) indicated that the effect of MAT on longevity is indirect and mediated through growth, whereas the direct effect of MAT on longevity was not significant (*P* = 0.1; Fig. 4c). Consistent with this result, the relationship between MAT and longevity became non-significant when growth was included as a covariate in phylogenetically informed regression (*P* = 0.49).

The strength of the growth–MAT relationship differed among life forms, being strongest in chamaephytes (*R*^2^ = 0.16, *P* < 0.0001), followed by hemicryptophytes (*R*^2^ = 0.15, *P* < 0.0001) and nanophanerophytes (*R*^2^ = 0.14, *P* < 0.0001), whereas lianas showed a weak association (*R*^2^ = 0.05, *P* = 0.02). In contrast, the longevity–MAT relationship was strongest in nanophanerophytes (*R*^2^ = 0.14, *P* < 0.0001), followed by chamaephytes (*R*^2^ = 0.11, *P* < 0.0001) and hemicryptophytes (*R*^2^ = 0.08, *P* < 0.0001), while lianas showed a non-significant association (*R*^2^ = 0, *P* = 0.7). When SEM was applied within life forms, the effect of MAT on longevity was largely indirect and mediated through growth, although a weak direct effect persisted in most groups (Supplementary Table 2). The only exception was lianas, for which no direct effect was detected. Consistent with these results, when growth rate was included as a covariate in phylogenetically informed models, the MAT–longevity relationship remained significant, although with substantially reduced effect sizes and higher *P*-values compared to models without growth. In lianas, however, the MAT–longevity relationship was not significant.

### Phylogenetic signal

Phylogenetic signal (*λ*) associated with the relationship between growth and longevity was generally moderate to high. Across all species analyzed, *λ* reached 0.8. Among different life forms, *λ* varied from 0.55 in lianas and hemicryptophytes to 0.26 in nanophanerophytes (Supplementary Table 1). Ecosystem-specific variation was also notable, with *λ* values as high as 0.7 in Arctic tundra and no detectable signal in cold desert–steppes (Supplementary Table 1).

Similarly, the overall phylogenetic signal for the relationship between growth and temperature was high (*λ* = 0.73), but varied among life forms: high in hemicryptophytes and chamaephytes (*λ* = 0.7), moderate in nanophanerophytes (*λ* = 0.16), and absent in lianas. For the relationship between longevity and temperature, the overall *λ* was 0.75, with lower values observed within specific life forms: lianas (0.7), chamaephytes (0.47), hemicryptophytes (0.39), and nanophanerophytes (0.22).

## Discussion

Using an extensive dataset encompassing more than 3100 species of hemicryptophytes, chamaephytes, nanophanerophytes, and lianas across ecosystems ranging from Arctic and alpine regions to temperate lowlands and hot semideserts, we demonstrate that high longevity is consistently associated with slow growth rates across the studied life forms and environments. While earlier studies have explored this relationship mainly at local scales^5,6,25^, and large-scale analyses have been predominantly limited to trees^7,12,13^, our results provide evidence that the growth–longevity trade-off extends consistently across diverse plant life forms and ecosystems, addressing a long-standing but largely untested assumption in plant ecological theory^9^. Furthermore, we show that plant longevity is linked to temperature, partly indirectly through its association with growth. This framework offers a basis for elucidating how life-history trade-offs may potentially affect plant responses in the context of ongoing climate change.

We found that the growth–longevity trade-off is remarkably robust, holding across all life forms and ecosystems studied. These findings are consistent with previous studies in herbaceous species that have identified growth–longevity trade-offs under specific environmental or taxonomic contexts^25,26^, as well as with analyses of demographic trade-offs across plant life forms^9,27^. This provides strong empirical support for the predictions derived from major ecological frameworks such as r–K selection theory, Grime’s CSR triangle, and the fast–slow plant economics spectrum, which posit that plants face fundamental allocation trade-offs between rapid growth to secure early advantages in light and nutrient acquisition and investment in stress tolerance that enhances long-term persistence at the expense of growth^15,16,19^. The trade-off was most pronounced in nanophanerophytes and chamaephytes, which maintain perennial, self-supporting above-ground organs (Fig. 2b and Supplementary Table 1). This pattern suggests that substantial investment in structural stability and mechanical endurance may amplify the growth–longevity trade-off in these life forms^35^, as slower growth can facilitate greater investment in dense, mechanically robust tissues^36,37^.

In contrast, the trade-off was weaker in hemicryptophytes, whose perennial organs are located below ground, partly buffered from environmental stresses such as wind, and in lianas, which rely on external mechanical support. Nevertheless, the persistence of significant relationships within these life forms indicates that additional mechanisms beyond biomechanics contribute to the growth–longevity trade-off. These may include allocation to carbon storage at the expense of growth, which can buffer plants against future unfavorable conditions and enhance stress tolerance^23^, as well as investment in chemical and structural defenses against pathogens and herbivores^22^. Moreover, the substantial phylogenetic signal detected in our analyses suggests that shared evolutionary history partly shapes the growth–longevity relationship, with closely related species tending to exhibit similar growth and longevity patterns^6^. Together, these results suggest that multiple, potentially interacting mechanisms underlie the association between growth and longevity, highlighting the need for integrative studies that disentangle their relative contributions and mechanistic bases.

We show that the relative abundance of fast-growing species increases with temperature. This intrinsic climate dependence aligns with experimental and observational findings that demonstrate growth acceleration under global warming and shifts in community composition favoring faster-growing species^26,29,38–40^. In parallel, our data also showed a negative association of longevity with temperature, a pattern previously observed in plant assemblages along elevational gradients^41^. Causal modeling suggests that this link is largely indirect, mediated by growth rate (Supplementary Table 2 and Fig. 4c). This underscores the significance of the growth–longevity association and offers a framework for understanding how life-history patterns develop across climatic gradients. These findings also suggest that climate change may influence the relative abundance of long-lived species in plant communities, which could be an important factor to consider when assessing impacts on vegetation^5,41^.

Although future warming could favor fast-growing species at the expense of long-lived species, we note that increasing drought stress may counteract this effect in many systems^30^. Regardless of direction, potential climate-driven changes in plant longevity are likely to have strong consequences for vegetation resilience to climate change^1^ and should therefore be incorporated into predictions of ecosystem responses. Importantly, our results highlight implications for terrestrial carbon sequestration. Shifts in plant longevity directly affect the residence time of carbon in living biomass, with potentially substantial consequences for the global carbon cycle^5^. While carbon sequestration has traditionally been considered primarily in the context of trees^4,42^, accumulating evidence shows that perennial herbs and shrubs also make substantial contributions^33,34^. The occurrence of exceptionally long-lived species across all life forms examined in this study underscores their capacity to store carbon in biomass over extended periods (Fig. 4d). We therefore propose that alterations in plant longevity represent a critical, yet underappreciated, component of future carbon-cycle projections.

While our results demonstrate that the growth–longevity relationship extends across plant life forms and diverse ecosystems, a substantial portion of the variation in longevity remained unexplained by growth alone. This is consistent with other large-scale studies reporting moderate or even context- and species-specific strengths of the trade-off^7,43^. Such patterns likely reflect the complexity of plant life-history strategies, which do not strictly conform to a single fast–slow continuum, as well as plants’ capacity to flexibly adjust resource allocation between growth, defense, and persistence in response to environmental conditions^9^. Together, these findings highlight that, although the growth–longevity trade-off is a pervasive axis of plant strategies, its expression is modulated by additional traits, environmental context, and plastic responses. Incorporating this multidimensionality in future research will be essential for improving predictions of plant longevity and, consequently, vegetation resilience under ongoing climate change.

Altogether, our findings complement previous research utilizing demographic metrics, such as survivorship curves and age at sexual maturity, to examine life-history trade-offs in several life forms, including herbs^9,27^. While these studies offer detailed insights into life-history strategies, our approach specifically addresses the growth–longevity trade-off across a significantly broader range of species and bioclimatic gradients, thereby complementing existing research by providing a more comprehensive generalization framework. Nonetheless, we acknowledge that certain regions, particularly the tropics, are underrepresented in our sampling. Recognizing this limitation, further research focused on these under-sampled environments is needed to validate and refine the patterns observed in our study. Additionally, we emphasize the significance of variability in longevity and growth across diverse ecosystems as a promising avenue for future studies investigating the diversity of life-history strategies^12^.

## Methods

### Plant collection

Plant material was collected by the authors and collaborators between 1994 and 2024 from natural, relatively undisturbed sites, as well as from botanic gardens and cultivated lands (~8% of species), primarily across the Northern Hemisphere. Sampling spanned a wide range of regions, including the Mongolian Gobi, the Himalayas (including Ladakh), the European Alps, the Japanese Alps, the Khangai Mountains, the U.S. Rockies, the Mojave, Sonora, Baja California, the Basin and Range, the Colorado Plateau, Western Tibet, Svalbard, and Greenland, among others. These efforts encompassed the environmental extremes of vascular plant life, ranging from the Sahara Desert to ~6150 m a.s.l. in the Ladakh Himalayas^26,44–46^, and to high Arctic sites in coastal East Greenland at ~73° N^47^.

The 3158 sampled species were classified into ecosystems, with 559 occurring in temperate montane ecosystems, 513 in temperate lowlands, 415 in Mediterranean montane ecosystems, 415 in Mediterranean scrubs, 331 in subalpine, 226 in alpine, 208 in warm semideserts, 190 in forest-steppe ecosystems, 139 in cold desert–steppes, 91 in Arctic tundra, and 71 in boreal forests.

All plants in our dataset are dicotyledonous angiosperms with secondary growth. Species were categorized as hemicryptophytes, with perennial below-ground structures bearing buds at ground level; chamaephytes, with buds on perennial shoots close to the ground; nanophanerophytes, with buds on perennial shoots positioned higher above the soil surface; and lianas, with buds on perennial above-ground shoots that rely on other plants for mechanical support. The most common life form among the studied species was hemicryptophytes (1986 species), followed by chamaephytes (620), nanophanerophytes (459), and lianas (93).

At each sampling site, adult individuals that appeared undamaged and healthy were selected for harvest. For each species, 5–10 of the tallest individuals were selected as they were expected to be the oldest, and their aboveground height was recorded, along with elevation and GPS coordinates. Small plants were carefully dug out to expose the root collar, whereas in shrubs, a disk at the root collar/base of the stem was cut with a saw, or a wood core was extracted with an increment borer (restricted to the main stem in multi-stemmed shrubs, as it is expected to be the oldest). Throughout the manuscript, we refer to all such sampling points simply as the root collar. The sampled material was stored in sealed plastic bags containing a few drops of 40% ethanol to prevent mold growth, which could interfere with accurate age determination. Sampling complied with applicable local regulations and institutional requirements.

In this study, we refer to species longevity as the age of the oldest observed living individual within a given species, consistent with previous usage across plant life forms^6,12–14,25^. The age was inferred from growth rings in existing, live tissue of the oldest plant part, the root collar (the transition zone between the shoot and root at the soil surface). Because herbaceous plants typically decompose rapidly after death, growth-ring analyses cannot reliably be applied to dead individuals; accordingly, we don’t estimate age at death, nor does our metric correspond to demographic life expectancy or maximum lifespan. However, even in trees, longevity is often inferred from living individuals^12–14^, enabling direct comparison of our results with recent studies.

Although a subset of samples originated from botanic gardens or cultivated settings, these represented a minor proportion of the dataset (~8% of species). Additional analyses excluding these samples yielded quantitatively similar results, indicating that their inclusion did not bias the main conclusions (Supplementary Table 3).

The dataset analysed in this study was assembled from measurements collected by the authors and collaborators between 1994 and 2024. Although parts of these measurements have previously been used in books and peer-reviewed articles addressing different research questions^25,47–54^, the species-level dataset underlying the present study has not been published previously and was assembled specifically for this study. These earlier publications provide additional anatomical descriptions, comparative analyses across habitats, and methodological background relevant to the present study.

### Anatomical analysis of growth and longevity

Cross-sections of root collars were prepared using a sledge microtome^55^, stained with a 1:1 mixture of Astra Blue and Safranin, and mounted in Canada Balsam. High-resolution images were then captured for measurement. Annual growth rates were calculated as the mean ring width measured along two radii in multiple individuals, while species longevity was estimated by counting the number of rings in the oldest individual^56^.

### Estimation of species’ climatic preferences

We characterized the general climatic preferences of the species based on occurrence data of the Global Biodiversity Information Facility^57^ and climate data of CHELSA V2.1^58,59^. GBIF coordinates of each species were filtered as follows: (1) *basisOfRecord* was “HUMAN_OBSERVATION”, “OCCURRENCE”, “OBSERVATION”, “MACHINE_OBSERVATION”, or “PRESERVED_SPECIMEN”, (2) the observation *year* was 1900 or later, (3) *coordinateUncertaintyInMeters* was below 10 km or missing, and (4) by applying CoordinateCleaner^60^ with the tests “capitals”, “centroids”, “equal”, “gbif”, “institutions”, “seas”, and “zeros”. Subsequently, the filtered coordinates were rasterized to the spatial reference of CHELSA (30 arc sec, ~1 km) using the R package terra^61^, and the centroid coordinates of each occupied cell were used for the extraction of mean annual temperature (i.e., bioclimatic variable bio1 in CHELSA) and mean annual precipitation (MAP, i.e., bio12). The two medians of the MAT and MAP values, extracted using the R package terra^61^, finally served as a rough characterization of the species’ climate preferences. GBIF occurrences at 30-arc-second resolution were aggregated to 1.0° species richness and mapped using the R packages ggplot2^62^ and maps^63^ to show the global coverage of the species considered (Fig. 3).

### Species phylogeny

Because species are evolutionarily related, they cannot be treated as independent observations in statistical models. To account for phylogenetic relatedness among the studied taxa, we constructed a phylogenetic tree using the U.PhyloMaker R package^64^. This package generates a phylogeny from a species list using a megatree as a backbone. For our analysis, we employed the *GBOTB.extended.WP.tre* megatree^65^.

### Data analysis

To assess the relationship between growth rate and longevity, we used phylogenetic linear models using the *pgls* function in the caper R package^66^ to account for shared evolutionary history among species. Phylogenetic signal (Pagel’s *λ*; 0 = no signal, 1 = Brownian motion model of evolution) was estimated by maximum likelihood. Both growth rate and longevity were log-transformed to reduce skewness and meet model assumptions. We first tested the simple relationship between growth and longevity, then examined whether this relationship varied across life forms by including an interaction term between growth rate and life form. Similarly, we tested for an interaction between growth rate and ecosystem. To assess differences in the slopes of the regression lines among life forms, we performed pairwise comparisons and used the Holm–Bonferroni method to control the family-wise error rate. To explore these patterns in greater detail, we next fitted separate models for each life form and ecosystem.

Additionally, we examined how growth and longevity are influenced by MAT both across the full dataset and within each life form. We also tested whether the association between longevity and MAT is direct or mediated indirectly through growth. To this end, we applied structural equation modeling (SEM) using the lavaan R package^67^. We specified a model in which MAT was included as a predictor of growth rate, and both MAT and growth rate were specified as predictors of plant longevity. This model structure was chosen to explicitly test whether the effect of temperature on longevity is direct, indirectly mediated through growth, or reflects a combination of both pathways (partial mediation). The indirect effect was quantified as the product of the MAT → growth and growth → longevity paths. Statistical significance of path coefficients was assessed using z-tests and associated *p*-values. Both longevity and growth were log-transformed, and all variables were standardized prior to analysis. Furthermore, we analyzed the relationship between longevity and MAT using phylogenetic linear models, including growth as a covariate, to assess whether the effect of MAT remained significant after accounting for growth. This analysis served as a phylogenetically informed complement to the SEM framework.

All analyses were conducted in R version 4.5.1^68^.

### Reporting summary

Further information on research design is available in the Nature Portfolio Reporting Summary linked to this article.

## Supplementary information

Supplementary Information

Reporting Summary

Transparent Peer Review file

## Data Availability

The complete processed species-level dataset necessary to reproduce all analyses presented in this study has been deposited in the Figshare repository and is available at https://doi.org/10.6084/m9.figshare.32288109. The deposited dataset contains the whole species-level database in a standardized form. Untransformed species-level measurements are not publicly available because they are currently being used in ongoing research projects. Requests for access to the complete database for non-commercial research purposes should be directed to the corresponding author. Access requests will be evaluated in consultation with the data contributors, and a response will normally be provided within four weeks. Species occurrence records used to characterize climatic preferences were obtained from the Global Biodiversity Information Facility (GBIF) https://doi.org/10.15468/dl.hbaee6. Climatic data were obtained from CHELSA V2.1 https://doi.org/10.5061/dryad.kd1d4.

## Code availability

R code used in this study has been deposited in the Figshare repository and is available at https://doi.org/10.6084/m9.figshare.32288109.
