# Supplementary material for: Slow growth as a strategy for plant longevity
Source: Nat Commun. 2026 Aug 13;17:9703. doi: 10.1038/s41467-026-76765-0 (PMC13562615; doi:10.1038/s41467-026-76765-0)
Supplement: Supplementary file 1 [file 41467_2026_76765_MOESM1_ESM.pdf]

Supplementary information for:

Slow growth as a strategy for plant longevity

Authors

Jan Binter[1, 2] *, Michael Peter Nobis[3], Jan Altman[1, 4], Ulf Büntgen[5, 6, 7], Alan Crivellaro[8, 9], Pierre Liancourt[10, 11] †, Jiří Doležal[1, 2] †

[1]Institute of Botany of the Czech Academy of Sciences, Průhonice, Czech Republic

[2]Faculty of Science, University of South Bohemia, České Budějovice, Czech Republic

[3]Swiss Federal Research Institute WSL, Birmensdorf, Switzerland

[4]Faculty of Forestry and Wood Sciences, Czech University of Life Sciences, Prague, Czech Republic

[5]Department of Geography, University of Cambridge, Cambridge, United Kingdom.

[6]Global Change Research Institute (CzechGlobe), Czech Academy of Sciences, Brno, Czech Republic.

[7]Department of Geography, Faculty of Science, Masaryk University, Brno, Czech Republic.

[8]Department of Agricultural, Forest and Food Sciences, Università degli Studi di Torino, Grugliasco, Torino, Italy.

[9]Forest Biometrics Laboratory, Faculty of Forestry, "Stefan cel Mare" University of Suceava, Suceava, Romania.

[10]Department of Botany, State Museum of Natural History Stuttgart, Stuttgart, Germany

[11]Plant Ecology Group, University of Tübingen, Tübingen, Germany

Contact information: jbinter30@gmail.com

Supplementary Table 1. Relationship between longevity and growth rates across distinct life forms and ecosystems. The table reports slope of regression line, R^2 and *p*-values, and the phylogenetic signal (Pagel's λ; 0 indicates no signal, 1 indicates a Brownian motion model). All parameters were estimated from phylogenetic models in which both variables were log-transformed. Analyses were based on *a priori* defined habitat and life-form categories; therefore, no correction for multiple comparisons was applied.

Category	Slope of regression line	Explained variability (R^2)	*p*-value	Lambda (λ)
Overall relationship	-0.40	0.22	$< 2.2 \times 10^{-16}$	0.80
Life form: Nanophanerophytes	-0.55	0.37	$< 2.2 \times 10^{-16}$	0.26
Life form: Chamaephytes	-0.56	0.34	$< 2.2 \times 10^{-16}$	0.51
Life form: Hemicryptophytes	-0.39	0.23	$< 2.2 \times 10^{-16}$	0.55
Life form: Lianas	-0.30	0.14	0.00017	0.55
Ecosystem: Arctic tundra	-0.48	0.15	7.4×10^{-5}	0.7
Ecosystem: Boreal forest	-0.33	0.06	0.03	0.67
Ecosystem: Alpine	-0.39	0.13	2.2×10^{-8}	0.63
Ecosystem: Subalpine	-0.33	0.09	1.3×10^{-8}	0.37
Ecosystem: Cold desert–steppe	-0.44	0.13	9.2×10^{-6}	0
Ecosystem: Warm semidesert	-0.5	0.43	$< 2.2 \times 10^{-16}$	0.61
Ecosystem: Mediterranean scrub	-0.38	0.18	$< 2.2 \times 10^{-16}$	0.38
Ecosystem: Mediterranean montane	-0.38	0.20	$< 2.2 \times 10^{-16}$	0.48
Ecosystem: Forest–Steppe	-0.40	0.17	2×10^{-9}	0.66
Ecosystem: Temperate montane	-0.33	0.13	$< 2.2 \times 10^{-16}$	0.6
Ecosystem: Temperate lowland	-0.33	0.18	$< 2.2 \times 10^{-16}$	0.65

Supplementary Table 2**.** Results of the structural equation models (SEM) testing the relationships among mean annual temperature (MAT), growth rate, and longevity. The table reports standardized path coefficients and associated *p*-values. The indirect effect of MAT on longevity mediated through growth was calculated as the product of the MAT → growth and growth → longevity paths. Analyses were based on *a priori* defined habitat and life-form categories; therefore, no correction for multiple comparisons was applied.

Life form	Predictor	Response variable	Path coefficient	*p*-value
Across life forms	MAT	Growth	0.48	$< 2.2 \times 10^{-16}$
Across life forms	MAT	Longevity	0.03	0.1
Across life forms	Growth	Longevity	-0.44	$< 2.2 \times 10^{-16}$
Across life forms	Indirect effect: MAT → Growth → Longevity		-0.21	$< 2.2 \times 10^{-16}$
Hemicryptophytes	MAT	Growth	0.45	$< 2.2 \times 10^{-16}$
Hemicryptophytes	MAT	Longevity	-0.1	$< 2.2 \times 10^{-16}$
Hemicryptophytes	Growth	Longevity	-0.44	$< 2.2 \times 10^{-16}$
Hemicryptophytes	Indirect effect: MAT → Growth → Longevity		-0.2	$< 2.2 \times 10^{-16}$
Chamaephytes	MAT	Growth	0.49	$< 2.2 \times 10^{-16}$
Chamaephytes	MAT	Longevity	-0.14	$< 2.2 \times 10^{-16}$
Chamaephytes	Growth	Longevity	-0.57	$< 2.2 \times 10^{-16}$
Chamaephytes	Indirect effect: MAT → Growth → Longevity		-0.28	$< 2.2 \times 10^{-16}$

Nanophanerophytes	MAT	Growth	0.43	$< 2.2 \times 10^{-16}$
Nanophanerophytes	MAT	Longevity	-0.23	$< 2.2 \times 10^{-16}$
Nanophanerophytes	Growth	Longevity	-0.55	$< 2.2 \times 10^{-16}$
Nanophanerophytes	Indirect effect: MAT → Growth → Longevity		-0.24	1.7×10^{-16}
Lianas	MAT	Growth	0.24	0.02
Lianas	MAT	Longevity	-0.003	0.97
Lianas	Growth	Longevity	-0.38	< 0.001
Lianas	Indirect effect: MAT → Growth → Longevity		-0.09	0.045

Supplementary Table 3. Relationship between longevity and growth rate, as well as between growth rate and mean annual temperature (MAT), excluding planted species. The table reports R^2 and *p*-values, as well as the phylogenetic signal (Pagel's *λ*; 0 indicates no signal, 1 indicates a Brownian motion model). Additionally, the table separately reports these relationships for planted specimens. All parameters were estimated from phylogenetic models in which growth and longevity were log-transformed. Analyses were based on *a priori* defined habitat and life-form categories; therefore, no correction for multiple comparisons was applied.

Longevity vs. Growth	Explained variability (R^2)	*p*-value	Lambda (*λ*)
Overall relationship	0.22	$< 2.2 \times 10^{-16}$	0.80
Life form: Nanophanerophytes	0.40	$< 2.2 \times 10^{-16}$	0.24
Life form: Chamaephytes	0.35	$< 2.2 \times 10^{-16}$	0.51
Life form: Hemicryptophytes	0.23	$< 2.2 \times 10^{-16}$	0.54
Life form: Lianas	0.10	0.007	0.41
Ecosystem: Arctic tundra	0.15	7.4×10^{-5}	0.7
Ecosystem: Boreal forest	0.05	0.04	0.69
Ecosystem: Alpine	0.13	2.9×10^{-8}	0.62
Ecosystem: Subalpine	0.08	3×10^{-7}	0.37
Ecosystem: Cold desert–steppe	0.13	1.6×10^{-5}	0
Ecosystem: Warm semidesert	0.42	$< 2.2 \times 10^{-16}$	0.61
Ecosystem: Mediterranean scrub	0.17	$< 2.2 \times 10^{-16}$	0.19
Ecosystem: Mediterranean montane	0.21	$< 2.2 \times 10^{-16}$	0.51
Ecosystem: Forest–Steppe	0.16	4.3×10^{-8}	0.62
Ecosystem: Temperate montane	0.13	$< 2.2 \times 10^{-16}$	0.6
Ecosystem: Temperate lowland	0.20	$< 2.2 \times 10^{-16}$	0.71
Planted specimens	0.08	3.9×10^{-6}	0.73
Growth vs. MAT	Explained variability (R^2)	*p*-value	Lambda (*λ*)
Overall relationship	0.17	$< 2.2 \times 10^{-16}$	0.18
Life form: Nanophanerophytes	0.17	$< 2.2 \times 10^{-16}$	0.18
Life form: Chamaephytes	0.16	$< 2.2 \times 10^{-16}$	0.7
Life form: Hemicryptophytes	0.15	$< 2.2 \times 10^{-16}$	0.71
Life form: Lianas	0.06	0.03	0
Planted specimens	0.06	3×10^{-5}	0.48
