# Supplementary material for: Slow growth as a strategy for plant longevity
Source: Nat Commun. 2026 Aug 13;17:9703. doi: 10.1038/s41467-026-76765-0 (PMC13562615; doi:10.1038/s41467-026-76765-0)
Supplement: Supplementary file 2 [file 41467_2026_76765_MOESM2_ESM.pdf]

nature portfolio

Corresponding author(s): Jan Binter
Last updated by author(s): Jul 14, 2026

Reporting Summary

Nature Portfolio wishes to improve the reproducibility of the work that we publish. This form provides structure for consistency and transparency in reporting. For further information on Nature Portfolio policies, see our Editorial Policies and the Editorial Policy Checklist.

Statistics

For all statistical analyses, confirm that the following items are present in the figure legend, table legend, main text, or Methods section.

n/a	Confirmed	
☐	☒	The exact sample size (*n*) for each experimental group/condition, given as a discrete number and unit of measurement
☐	☒	A statement on whether measurements were taken from distinct samples or whether the same sample was measured repeatedly
☐	☒	The statistical test(s) used AND whether they are one- or two-sided<br>*Only common tests should be described solely by name; describe more complex techniques in the Methods section.*
☐	☒	A description of all covariates tested
☐	☒	A description of any assumptions or corrections, such as tests of normality and adjustment for multiple comparisons
☐	☒	A full description of the statistical parameters including central tendency (e.g. means) or other basic estimates (e.g. regression coefficient) AND variation (e.g. standard deviation) or associated estimates of uncertainty (e.g. confidence intervals)
☐	☒	For null hypothesis testing, the test statistic (e.g. *F*, *t*, *r*) with confidence intervals, effect sizes, degrees of freedom and *P* value noted<br>*Give P values as exact values whenever suitable.*
☒	☐	For Bayesian analysis, information on the choice of priors and Markov chain Monte Carlo settings
☒	☐	For hierarchical and complex designs, identification of the appropriate level for tests and full reporting of outcomes
☒	☐	Estimates of effect sizes (e.g. Cohen's *d*, Pearson's *r*), indicating how they were calculated

Our web collection on statistics for biologists contains articles on many of the points above.

Software and code

Policy information about availability of computer code

Data collection	We characterized the general climatic preferences of the species based on occurrence data of the Global Biodiversity Information Facility and climate data of CHELSA V2.1.
Data analysis	All analyses were conducted in R version 4.5.1. The R packages used were caper, v1.0.3; U.PhyloMaker, v0.1.0; lavaan, v0.6-21; terra, v1.8-70; maps, v3.4.3.

For manuscripts utilizing custom algorithms or software that are central to the research but not yet described in published literature, software must be made available to editors and reviewers. We strongly encourage code deposition in a community repository (e.g. GitHub). See the Nature Portfolio guidelines for submitting code & software for further information.

Data

Policy information about availability of data

All manuscripts must include a data availability statement. This statement should provide the following information, where applicable:

- Accession codes, unique identifiers, or web links for publicly available datasets
- A description of any restrictions on data availability
- For clinical datasets or third party data, please ensure that the statement adheres to our policy

The complete processed species-level dataset necessary to reproduce all analyses presented in this study has been deposited in the Figshare repository and is available at https://doi.org/10.6084/m9.figshare.32288109. The deposited dataset contains the whole species-level database in a standardized form. Untransformed species-level measurements are not publicly available because they are currently being used in ongoing research projects. Requests for access to

the complete database for non-commercial research purposes should be directed to the corresponding author. Access requests will be evaluated in consultation with the data contributors, and a response will normally be provided within four weeks.
Species occurrence records used to characterize climatic preferences were obtained from the Global Biodiversity Information Facility (GBIF; https://doi.org/10.15468/dl.hbaee6). Climatic data were obtained from CHELSA V2.1 (https://doi.org/10.5061/dryad.kd1d4).

Research involving human participants, their data, or biological material

Policy information about studies with human participants or human data. See also policy information about sex, gender (identity/presentation), and sexual orientation and race, ethnicity and racism.

Reporting on sex and gender	*Use the terms sex (biological attribute) and gender (shaped by social and cultural circumstances) carefully in order to avoid confusing both terms. Indicate if findings apply to only one sex or gender; describe whether sex and gender were considered in study design; whether sex and/or gender was determined based on self-reporting or assigned and methods used. Provide in the source data disaggregated sex and gender data, where this information has been collected, and if consent has been obtained for sharing of individual-level data; provide overall numbers in this Reporting Summary. Please state if this information has not been collected. Report sex- and gender-based analyses where performed, justify reasons for lack of sex- and gender-based analysis.*
Reporting on race, ethnicity, or other socially relevant groupings	*Please specify the socially constructed or socially relevant categorization variable(s) used in your manuscript and explain why they were used. Please note that such variables should not be used as proxies for other socially constructed/relevant variables (for example, race or ethnicity should not be used as a proxy for socioeconomic status). Provide clear definitions of the relevant terms used, how they were provided (by the participants/respondents, the researchers, or third parties), and the method(s) used to classify people into the different categories (e.g. self-report, census or administrative data, social media data, etc.) Please provide details about how you controlled for confounding variables in your analyses.*
Population characteristics	*Describe the covariate-relevant population characteristics of the human research participants (e.g. age, genotypic information, past and current diagnosis and treatment categories). If you filled out the behavioural & social sciences study design questions and have nothing to add here, write "See above."*
Recruitment	*Describe how participants were recruited. Outline any potential self-selection bias or other biases that may be present and how these are likely to impact results.*
Ethics oversight	*Identify the organization(s) that approved the study protocol.*

Note that full information on the approval of the study protocol must also be provided in the manuscript.

Field-specific reporting

Please select the one below that is the best fit for your research. If you are not sure, read the appropriate sections before making your selection.

☐ Life sciences ☐ Behavioural & social sciences ☒ Ecological, evolutionary & environmental sciences

For a reference copy of the document with all sections, see nature.com/documents/nr-reporting-summary-flat.pdf

Ecological, evolutionary & environmental sciences study design

All studies must disclose on these points even when the disclosure is negative.

Study description	This study examines the relationship between growth and longevity in 3,158 perennial plant species, including 1,986 hemicryptophytes, 620 chamaephytes, 459 nanophanerophytes, and 93 lianas from a wide range of ecosystems. We further assess the relationship of these variables with temperature, providing insights into potential shifts in plant lifespan and carbon dynamics under global warming.
Research sample	All plants in our dataset are dicotyledonous angiosperms, including hemicryptophytes, chamaephytes, nanophanerophytes, and lianas. Species were selected to capture diverse life forms and a broad range of ecosystems. For each species, 5–10 of the tallest, healthy individuals were sampled, as these were expected to represent the oldest mature individuals of the species. The study aims to characterize species-level growth and longevity across environmental gradients rather than demographic variation within populations.
Sampling strategy	For each species, 5–10 of the tallest, healthy individuals were sampled using a targeted rather than random sampling design. No statistical methods were used to predetermine sample size, as the objective was not to estimate population-level demographic parameters but to identify the oldest living individuals of each species. Sampling 5–10 of the largest individuals was considered sufficient to provide a robust estimate of species longevity while remaining feasible across the large number of species included in the study.
Data collection	Plant material was collected between 1994 and 2024 from natural, relatively undisturbed sites, as well as from botanic gardens and cultivated lands. For each species, 5–10 of the tallest healthy individuals were selected for sampling. In small plants, the root collar was exposed by careful excavation, whereas in shrubs samples were obtained either by cutting a stem disc or by extracting a wood core. All field data were recorded directly by the authors and collaborators. Geographic coordinates were recorded using handheld

	GPS devices, and all other field measurements were recorded manually with pen and paper. Although parts of these measurements have previously been used in books and peer-reviewed articles addressing different research questions, the species-level dataset underlying the present study has not been published previously and was assembled specifically for this study.
Timing and spatial scale	The sampling was conducted over the past three decades (1994–2024), with field campaigns organized during the growing seasons. This long-term effort ensured consistent coverage of species, life forms, and environmental conditions. Sampling spanned regions worldwide, including the Mongolian Gobi, Himalayas (Ladakh), European and Japanese Alps, Khangai Mountains, US Rockies, Mojave and Sonora Deserts, Baja California, the Basin and Range, Colorado Plateau, Western Tibet, Svalbard, and Greenland, among others.
Data exclusions	We excluded three species whose climatic preferences represented severe outliers compared with the remainder of the dataset. Importantly, this decision was taken independently of the study's outcomes and reflects a precautionary step to ensure robust and representative analyses.
Reproducibility	Reproducibility was ensured by the large sample size and by the consistent application of standardized procedures across all species.
Randomization	All measurements were obtained from naturally occurring plants in their native habitats or from individuals growing in botanical gardens and cultivated lands. As no experimental manipulation was involved, allocation of samples into groups was not applicable.
Blinding	Because all measurements were taken directly from naturally occurring plants in their habitats, or from individuals in botanical gardens and cultivated lands, blinding was neither feasible nor relevant to this study.

Did the study involve field work? [x] Yes [ ] No

Field work, collection and transport

Field conditions	By sampling species with broad climatic optima, we achieved coverage of diverse regimes spanning contrasting environmental conditions.
Location	Sampling spanned regions worldwide, including the Mongolian Gobi, Himalayas (Ladakh), European and Japanese Alps, Khangai Mountains, US Rockies, Mojave and Sonora Deserts, Baja California, the Basin and Range, Colorado Plateau, Western Tibet, Svalbard, and Greenland, among others.
Access & import/export	Sampling was conducted in accordance with applicable local and national regulations. Given the temporal (1994–2024) and geographic extent of the dataset, individual permission records are not reported.
Disturbance	To minimize ecological impact, only the necessary amount of plant material was collected, with all sampling conducted in a way that ensured minimal disturbance to the ecosystem.

Reporting for specific materials, systems and methods

We require information from authors about some types of materials, experimental systems and methods used in many studies. Here, indicate whether each material, system or method listed is relevant to your study. If you are not sure if a list item applies to your research, read the appropriate section before selecting a response.

Materials & experimental systems

n/a	Involved in the study	
[x]	[ ]	Antibodies
[x]	[ ]	Eukaryotic cell lines
[x]	[ ]	Palaeontology and archaeology
[x]	[ ]	Animals and other organisms
[x]	[ ]	Clinical data
[x]	[ ]	Dual use research of concern
[ ]	[x]	Plants

Methods

n/a	Involved in the study	
[x]	[ ]	ChIP-seq
[x]	[ ]	Flow cytometry
[x]	[ ]	MRI-based neuroimaging

Plants

Seed stocks

No seed stocks were used; all plant material was obtained either from wild-growing individuals or from plants maintained in botanical gardens and cultivated lands. Fieldwork was conducted over the past 30 years across a wide range of ecosystems worldwide. For each species, 5–10 of the tallest, healthy individuals were selected. In small plants, the root collar was carefully exposed by excavation, whereas in shrubs samples were obtained either by cutting a stem disk with a saw or by extracting a wood core.

Novel plant genotypes

Describe the methods by which all novel plant genotypes were produced. This includes those generated by transgenic approaches, gene editing, chemical/radiation-based mutagenesis and hybridization. For transgenic lines, describe the transformation method, the number of independent lines analyzed and the generation upon which experiments were performed. For gene-edited lines, describe the editor used, the endogenous sequence targeted for editing, the targeting guide RNA sequence (if applicable) and how the editor was applied.

Authentication

Describe any authentication procedures for each seed stock used or novel genotype generated. Describe any experiments used to assess the effect of a mutation and, where applicable, how potential secondary effects (e.g. second site T-DNA insertions, mosiacism, off-target gene editing) were examined.
