# Supplementary material for: Slow growth as a strategy for plant longevity
Source: Nat Commun. 2026 Aug 13;17:9703. doi: 10.1038/s41467-026-76765-0 (PMC13562615; doi:10.1038/s41467-026-76765-0)
Supplement: Supplementary file 3 [file 41467_2026_76765_MOESM3_ESM.pdf]

nature portfolio

Peer Review File

Slow growth as a strategy for plant longevity

Corresponding Author: Mr Jan Binter

This file contains all reviewer reports in order by version, followed by all author rebuttals in order by version.

Version 0:

Reviewer comments:

Reviewer #1

(Remarks to the Author)
The manuscript by Binter and co-authors assesses the nature of the growth-longevity trade-off across plants' life forms, other than trees. This trade-off has been widely reported across various biological systems, including plants. In the latter, it has been mostly reported in trees, at different scales, from global to interspecific variability. Nonetheless, there are just pieces of evidence for other life-forms, and to my knowledge, no global assessment has been conducted so far. Building on this knowledge gap, the authors bring a novel and timely study on this trade-off assessment based on herbaceous and small woody species. The dataset is rather impressive, with over 3000 species sampled across five continents. No question the studies hold a high scientific value and does engage on a clear and important research question.

The study also benefits from a clear and simple message: yes, the trade-off is also found in other life-forms other than trees. And I agree it has important ecological and biological implications.

Overall, the study is well-written. I just have some minor suggestions regarding the text. Please see some comments and suggestions below. And the methods have been clearly described, well thought and employed.

Although the study holds great value, no question, I believe some important issues must be addressed before it is found acceptable for publication here or in another outlet.

One gripe I have with such global datasets and studies is the claim of "universal" patterns. Believe me, I've been in your shoes, and I know how tempting it ia to communicate like this, and no question it is catchy. Still, despite the impressive dataset, there are clear gaps in the dataset coverage that prevent one from stating the study as global and universal. The tropics is largely underrepresented in the dataset, and although not so important in terms of herbaceous and shrubs, it holds the highest diversity of lianas. Other than lianas, the savannas also hold a relevant assembly of shrubs and herbaceous species that are not covered by the extensive dataset of this study. Does it reduce the value of the study? Clearly not, but this should be included in a paragraph discussing the study's limitations. And also, calls for a tone down in the title, abstract, and text.

The second issue I had with this nice study is the lack of additional information / results on some key analyses. Like, the phylogenetic analysis is indeed well employed in the study, but there is no report on the strength of the phylogenetic signal on the results. I know that it is supposed to be really high. Higher than any other environmental factors. This should be reported in depth in the manuscript.

Still regarding the methods, I am a little bit uncomfortable about the use of planted specimens in the study. It is not clear to me how using them could bias the results. It is worth checking if you have any bias, and then reporting it in the main text.

Finally, you do present a theoretical model to explain how temperature affects longevity. Why don’t you apply Structural Equation Models to test it?

Minor comments:

L26-41: At first, I thought the study did not cover an important life form, lianas. I was happy to see that lianas are included

further below. Please mention it in the abstract too.

L46: Don't think you need the "however here".

L59: resources to tolerate stress

L60: Perhaps you can say that the reduced growth rate is associated with. Because it is nearly impossible to know if there is a cause-and-effect relationship here, or if it is a common modulation by trees' metabolism and physiology.

L62: Perhaps, remove the word "collectively"

L66: trees consitute a methdological powerful model, or tree rings constitute it?

Figure 2: It took me a while to find the liana in panel B. Perhaps, you could also use dotted lines to connect the life forms and the pie chart, as for the lianas.

Figure. 4: Why not apply a Structural Equation Model instead of presenting it as a conceptual model?

L280: angiosperms with secondary growth

Reviewer #2

(Remarks to the Author)
Comments for the authors:
This manuscript tests classic assumptions about demographic trade-offs for herbaceous plants, using an impressive global dataset of more than 3,000 species. It shows a strong association between mean annual growth and mature plant age. The paper is very interesting, with clear novelty that will be of interest to a broad audience.That said, the paper would benefit from a bit more clarity in the terminology and a more rounded discussion of how these results relate to what is already known from comparative demographic studies of herbaceous species. I offer the following suggestions for refinement.

The term “longevity” seems misleading given what is actually being measured. This is a broad term that is often quantified using metrics such as mean life expectancy or maximal age at death (maximal lifespan). These terms have specific demographic meanings and would require more formal demographic analyses to estimate. Life expectancy is the mean age at death, and maximal lifespan is the maximum age at death. As I understand it, age here is estimated from a subset of healthy, mature, living plants. In that case, age at death is not being measured directly. To be clear, I think this is still a very interesting and meaningful variable; my concern is with the terminology, not the measurement itself. A term such as “mature plant age” seems more accurate and better aligned with what is actually being measured. It would also be more consistent with previous work comparing mature plant size or age with growth. Journé et al. (https://doi.org/10.1111/ele.14500) may be a useful example in this regard. Although the questions differ, the terminology and demographic measures are closely related, especially with respect to the link between age at maturity and life expectancy. It may also be useful to compare this framing with classic demographic concepts such as age at sexual maturity and its relationship to growth; see https://doi.org/10.1073/pnas.1506215112. This could help show that the metric used here may serve as a proxy for how long species can live.
I would be more cautious in stating that demographic trade-offs have rarely been examined in herbaceous species, for example in lines 29–30 and elsewhere in the Introduction. From a demographic perspective, there is already a substantial body of theory and empirical work on life-history trade-offs in herbaceous plants, some of which is already cited. To me, the main novelty of this paper is not that it reveals entirely unexplored patterns, but that it demonstrates these patterns across a much broader range of species and bioclimatic gradients. The manuscript would be strengthened by more explicitly highlighting both the similarities to, and differences from, previous work. Two relevant examples already cited are https://doi.org/10.1073/pnas.1506215112 and https://doi.org/10.1111/nph.14289. The manuscript already does a good job emphasizing its novelty, but a brief discussion of similarities and differences with earlier work would make the framing stronger.

It could be interesting to emphasize not only mean differences in growth and mature plant age across climate gradients, but also the diversity of strategies across those gradients. Macroecological theory suggests that the diversity of demographic strategies should vary systematically along climate gradients, with stronger abiotic filtering in cold or dry systems leading to a narrower range of life-history strategies that can persist under climatic extremes, and a wider range of strategies in temperate and tropical regions. One way to examine this would be to look at variance, rather than focusing only on mean values, across climate conditions. This is only a suggestion, but I think it could help connect the study to macroecological theory and to emerging patterns in the literature on trees, for example https://doi.org/10.1073/pnas.2003873117 (see Fig. 2), as well as other papers already cited in the manuscript.

Finally, I would be a bit more cautious in discussing the relationship between temperature and growth in ways that imply how species will respond to rapid climate change. The present results seem more naturally interpreted as evidence of broad biogeographic patterns in mean growth and in the diversity of growth strategies under current climate conditions. These patterns are likely shaped by long-term evolutionary processes, local adaptation, and abiotic filtering, rather than directly demonstrating shifts in species-level demography due to climate change. This is a relatively minor point, but I think a slightly

more restrained interpretation would improve the framing.

Comment on the Results: I did not fully understand the interpretation of “mediated by temperature” (around line 168). If the goal is to estimate a mediated effect, why not use a structural equation model, as Fig. 4 seems to imply visually? I may simply be missing something, but this point would benefit from clearer explanation.

Comment on the Abstract:“Pace of life” is a broad term that captures the timing of events across the life cycle, including growth strategies, age and size at maturity, and life expectancy, rather than referring specifically to life expectancy. I would suggest a small edit here for clarity.
Overall, my comments are mostly higher-level and concern terminology and framing rather than the core results. With some refinement in terminology and a more explicit connection to existing life-history theory, I think this impressive study will be of broad interest.

Version 1:

Reviewer comments:

Reviewer #1

(Remarks to the Author)
I have now read the new version of the manuscript. I believe it has benefited from the comments and suggestions of both reviewers, and I have no reservations in recommending it for publication.

Reviewer #2

(Remarks to the Author)
I commend the authors for their thoughtful revision. They have addressed the main comments and concerns I raised in the previous round, and I have no further comments. I believe this paper will be of interest to a broad audience.

Open Access This Peer Review File is licensed under a Creative Commons Attribution 4.0 International License, which permits use, sharing, adaptation, distribution and reproduction in any medium or format, as long as you give appropriate credit to the original author(s) and the source, provide a link to the Creative Commons license, and indicate if changes were made.
In cases where reviewers are anonymous, credit should be given to 'Anonymous Referee' and the source.
The images or other third party material in this Peer Review File are included in the article’s Creative Commons license, unless indicated otherwise in a credit line to the material. If material is not included in the article’s Creative Commons license and your intended use is not permitted by statutory regulation or exceeds the permitted use, you will need to obtain permission directly from the copyright holder.
To view a copy of this license, visit https://creativecommons.org/licenses/by/4.0/

We would like to thank the referees for the constructive and insightful feedback on our manuscript (NCOMMS-26-009954), entitled "*Slow growth as a strategy for plant longevity*", submitted to *Nature Communications*. The comments were extremely helpful and have substantially improved the clarity and overall quality of our work.

Following the referees' suggestions, we have made a number of revisions and clarifications. Below, we provide a summary of the main changes, followed by a detailed point-by-point response to each comment. Line numbers refer to the tracked-changes version of the revised manuscript.

1. **Universality of the observed patterns**
   We have revised the wording throughout the manuscript, including the title, to avoid overstatements of "universal" or "global" patterns. We now use more precise terms such as "widespread" and explicitly refer to associations across the studied life forms and ecosystems. We also acknowledge the underrepresentation of tropical regions in our dataset and discuss this limitation in the Discussion, highlighting the need for future work to address this gap.
2. **Phylogenetic signal**
   We now include a dedicated paragraph in the Results section reporting the strength of the phylogenetic signal in the fitted relationships. These results confirm that phylogenetic structure represents a major axis of variation underlying the observed patterns and are further integrated into the Discussion to better contextualize our findings.
3. **Inclusion of planted species**
   We now clarify that only a small proportion of the dataset (approximately 8%) consists of species from planted settings. To assess potential bias, we repeated the main analyses excluding these species, and additionally conducted analyses restricted to planted species only. In all cases, the results remained unchanged. These analyses demonstrate that the inclusion of planted individuals does not bias our conclusions.
4. **Structural equation modelling**
   We now employ structural equation modelling (SEM) to explicitly test whether growth mediates the effect of mean annual temperature (MAT) on longevity. This approach allows us to disentangle direct and indirect pathways and provides a formal evaluation of the proposed relationships. The results confirm our original interpretation and strengthen the mechanistic framework of the study.
5. **Use and definition of "longevity"**
   We now explicitly define species longevity as the age of the oldest observed living individual in our dataset within a given species. We clarify that this metric does not represent demographic life expectancy or true maximum lifespan, but instead serves as an empirical proxy for the upper ages attained by mature individuals. We also refer to previous work using similar definitions of the term. We apply this definition consistently throughout the manuscript and avoid interchangeable use of the term "lifespan." We have also removed the term "pace of life" to improve terminological clarity.
6. **Connection to existing literature**
   We have revised the manuscript to more clearly acknowledge that growth–longevity trade-offs have previously been documented in herbaceous species. We now emphasize that the primary contribution of our study lies in demonstrating this pattern across a substantially broader range of species and bioclimatic gradients. We also expand the Discussion to more explicitly address similarities to and differences from previous demographic studies.

7. **Diversity of strategies across ecosystems**
   We appreciate this suggestion and agree that examining the diversity of life-history strategies across environmental gradients is an important direction for future research. While this was beyond the scope of the present study, we now briefly acknowledge this perspective in the Discussion.
8. **Interpretation of climate change implications**
   We have revised the manuscript to adopt a more cautious interpretation of climate-related implications. We now emphasize that the observed patterns likely reflect long-term evolutionary processes, local adaptation, and abiotic filtering across climatic gradients. Accordingly, we avoid direct inference about species-level demographic responses to ongoing climate change and instead frame our results as indicative of potential shifts in species composition and associated changes in plant longevity.

Reviewer #1

One gripe I have with such global datasets and studies is the claim of "universal" patterns. Believe me, I've been in your shoes, and I know how tempting it ia to communicate like this, and no question it is catchy. Still, despite the impressive dataset, there are clear gaps in the dataset coverage that prevent one from stating the study as global and universal. The tropics is largely underrepresented in the dataset, and although not so important in terms of herbaceous and shrubs, it holds the highest diversity of lianas. Other than lianas, the savannas also hold a relevant assembly of shrubs and herbaceous species that are not covered by the extensive dataset of this study. Does it reduce the value of the study? Clearly not, but this should be included in a paragraph discussing the study's limitations. And also, calls for a tone down in the title, abstract, and text.

Response: We thank the reviewer for this important and thoughtful comment. We agree that our original wording overstated the generality of the observed patterns and that claims of universality are not justified given current sampling limitations. We have therefore revised the manuscript throughout, including the title, abstract, and main text, to avoid terms such as “universal” or “global.” Instead, we now describe the observed relationships as “widespread” and explicitly refer to their occurrence across the studied life forms and ecosystems.

We also acknowledge that tropical regions, including liana-rich systems and savanna ecosystems, are underrepresented in our dataset. This limitation is now explicitly discussed in the Discussion (L334–337), where we highlight the need for future work to address these under-sampled regions and to test the generality of the observed patterns more comprehensively.

The second issue I had with this nice study is the lack of additional information / results on some key analyses. Like, the phylogenetic analysis is indeed well employed in the study, but there is no report on the strength of the phylogenetic signal on the results. I know that it is supposed to be really high. Higher than any other environmental factors. This should be reported in depth in the manuscript.

Response: We thank the reviewer for this important point. While phylogenetic signal was originally included in the Supplementary Information, we agree that it was not sufficiently highlighted in the main text. We have now added a dedicated paragraph in the Results (L230–242) reporting the strength of the phylogenetic signal for the growth–longevity, growth–temperature, and longevity–temperature relationships. These results confirm that phylogenetic structure represents a major axis of variation underlying the observed relationships.

We further incorporate these results into the Discussion (L279–281) to better contextualize the role of phylogenetic structure in shaping the observed patterns.

Still regarding the methods, I am a little bit uncomfortable about the use of planted specimens in the study. It is not clear to me how using them could bias the results. It is worth checking if you have any bias, and then reporting it in the main text.

Response: We thank the reviewer for raising this concern. We now clarify that only a small proportion of the dataset (approximately 8%) consists of species from botanical gardens or cultivated settings (L344, from L383).

To assess potential bias, we repeated the main analyses excluding these species, including the growth–longevity relationships across life forms and ecosystems, as well as the relationships between growth and mean annual temperature. In all cases, the results remained unchanged. We also conducted analyses restricted to cultivated species only, which yielded consistent patterns.

These analyses demonstrate that inclusion of planted individuals does not bias the observed relationships. These findings are summarized in the main text (from L383) and reported in detail in Supplementary Table 3.

Finally, you do present a theoretical model to explain how temperature affects longevity. Why don't you apply Structural Equation Models to test it?

Response: We thank the reviewer for this valuable suggestion. In the original analysis, we assessed the potential mediation of the temperature–longevity relationship by including growth as a covariate in the longevity–temperature model, which allowed us to evaluate whether the association between temperature and longevity persisted after accounting for growth.

Following the reviewer's suggestion, we have now additionally applied structural equation modelling (SEM) to explicitly test whether the effect of mean annual temperature (MAT) on longevity is direct or mediated through growth. The SEM results are fully consistent with our original approach and confirm the same causal interpretation.

These results are now presented in the Results section (from L192), in Supplementary Table 2, and in a revised Figure 4, which now includes a path diagram with standardized coefficients.

L26-41: At first, I thought the study did not cover an important life form, lianas. I was happy to see that lianas are included further below. Please mention it in the abstract too.

Response: We have revised the sentence accordingly to improve clarity.

L46: Don't think you need the "however here".

Response: We removed "however".

L59: resources to tolerate stress

Response: We have revised the text accordingly.

L60: Perhaps you can say that the reduced growth rate is associated with. Because it is nearly impossible to know if there is a cause-and-effect relationship here, or if it is a common modulation by trees' metabolism and physiology.

Response: We thank the reviewer for this important clarification. We have revised the text to describe this relationship as an association rather than a causal effect, improving conceptual accuracy.

L62: Perhaps, remove the word "collectively"

Response: We have removed the word "collectively".

L66: trees consitute a methdological powerful model, or tree rings constitute it?

Response: In this case, we mean that trees constitute powerful model organisms. We have revised the text to improve clarity.

Figure 2: It took me a while to find the liana in panel B. Perhaps, you could also use dotted lines to connect the life forms and the pie chart, as for the lianas.

Response: We have revised the figure by improving the visibility of lianas and adding connecting lines for clarity.

Figure. 4: Why not apply a Structural Equation Model instead of presenting it as a conceptual model?

Response: We thank the reviewer for this valuable suggestion. Following this comment, we have replaced the conceptual model with a formal structural equation modelling (SEM) analysis to explicitly test the hypothesized relationships among temperature, growth, and longevity. The SEM allows us to disentangle direct and indirect effects and provides a quantitative evaluation of the proposed causal pathways. The results are consistent with our initial conceptual framework and support the same interpretation. The SEM is now presented in the Results section (from L192), in Supplementary Table 2, and in a revised Figure 4 including a path diagram with standardized coefficients.

L280: angiosperms with secondary growth

Response: We have revised the text accordingly.

Reviewer #2

The term "longevity" seems misleading given what is actually being measured. This is a broad term that is often quantified using metrics such as mean life expectancy or maximal age at death (maximal lifespan). These terms have specific demographic meanings and would require more formal demographic analyses to estimate. Life expectancy is the mean age at death, and maximal lifespan is the maximum age at death. As I understand it, age here is estimated from a subset of healthy, mature, living plants. In that case, age at death is not being measured directly. To be clear, I think this is still a very interesting and meaningful variable; my concern is with the terminology, not the measurement itself. A term such as "mature plant age" seems more accurate and better aligned with what is actually being measured. It would also be more consistent with previous work comparing mature plant size or age with growth. Journé et al. (https://doi.org/10.1111/ele.14500) may be a useful example in this regard. Although the questions differ, the terminology and demographic measures are closely related, especially with respect to the link between age at maturity and life expectancy. It may also be useful to compare this framing with classic demographic concepts such as age at sexual maturity and its relationship to growth; see https://doi.org/10.1073/pnas.1506215112. This could help show that the metric used here may serve as a proxy for how long species can live.

Response: We thank the reviewer for this important and thoughtful comment. We agree that the term “longevity” can encompass different demographic meanings, and that estimation of mean life expectancy or maximum age at death would require formal demographic data.

In this study, we do not estimate age at death, and we fully acknowledge that our metric does not correspond to demographic life expectancy or true maximum lifespan. We therefore clarify that we define species longevity as the age of the oldest observed living individual in our dataset within a given species (now specified in L118–120; from L374). Specifically, for each species, we sampled 5–10 of the largest individuals (expected to be the oldest) and determined their age from growth rings in the oldest living tissue (root collar). The age of the oldest individual was then taken as an estimate of species longevity. This approach reflects methodological constraints, as herbaceous plants typically decompose rapidly after death and growth-ring analyses cannot reliably be applied to dead individuals.

While we appreciate the suggestion to use the term “mature plant age,” we retain the term “longevity” to reflect our targeted sampling of the largest individuals (expected to be the oldest; L366) and the focus on maximum observed age within species. Similar definitions have been used in previous studies (Chondol et al., 2023; Gao et al., 2025; Locosselli et al., 2025; Samraoui et al., 2025), including recent work published in this journal (Brienen et al., 2025), now cited in the main text (L118–120; from L374).

We acknowledge that the term was not clearly defined in the key parts of the manuscript. To avoid ambiguity, we now explicitly define it in the Introduction (L118–120), with a more detailed explanation provided in the Methods (from L374). We also use “longevity” consistently throughout the manuscript, avoiding interchangeable use with “lifespan.”

We further acknowledge that demographic metrics such as age at sexual maturity and survivorship-based measures are closely linked to growth. To connect our approach to this framework, we now directly compare our results with studies such as Salguero-Gómez et al. (2015), which demonstrate coordinated relationships among these demographic variables and their inverse association with growth (L259-262; from L329). This supports the interpretation of our metric as a proxy for species-level differences in longevity.

Overall, we have revised the manuscript to clearly define the term “longevity” and to better position our metric within the broader demographic framework.

I would be more cautious in stating that demographic trade-offs have rarely been examined in herbaceous species, for example in lines 29–30 and elsewhere in the Introduction. From a demographic perspective, there is already a substantial body of theory and empirical work on life-history trade-offs in herbaceous plants, some of which is already cited. To me, the main novelty of this paper is not that it reveals entirely unexplored patterns, but that it demonstrates these patterns across a much broader range of species and bioclimatic gradients.

Response: We thank the reviewer for this important clarification. We agree that our original wording did not sufficiently acknowledge the existing body of work on life-history trade-offs in herbaceous species.

We have revised the Introduction and Discussion to explicitly recognize that demographic trade-offs have been widely studied in herbaceous plants, including studies spanning multiple ecosystems and life forms, while noting that many analyses remain constrained to particular taxa or environmental contexts (from L77; L259–262).

We now frame the novelty of our study more precisely as demonstrating the growth–longevity relationship across a substantially broader range of species and bioclimatic gradients.

The manuscript would be strengthened by more explicitly highlighting both the similarities to, and differences from, previous work. Two relevant examples already cited are https://doi.org/10.1073/pnas.1506215112 and https://doi.org/10.1111/nph.14289. The manuscript already does a good job emphasizing its novelty, but a brief discussion of similarities and differences with earlier work would make the framing stronger.

Response: We thank the reviewer for raising this important point. To better situate our findings within the existing literature, we have expanded the Discussion to explicitly compare our results with previous studies (L259–262). We further address similarities and differences relative to large-scale demographic studies exploring life-history trade-offs across plant life forms and ecosystems (from L329). We highlight how our findings complement this body of work while emphasizing the novelty of the present study.

It could be interesting to emphasize not only mean differences in growth and mature plant age across climate gradients, but also the diversity of strategies across those gradients. Macroecological theory suggests that the diversity of demographic strategies should vary systematically along climate gradients, with stronger abiotic filtering in cold or dry systems leading to a narrower range of life-history strategies that can persist under climatic extremes, and a wider range of strategies in temperate and tropical regions. One way to examine this would be to look at variance, rather than focusing only on mean values, across climate conditions. This is only a suggestion, but I think it could help connect the study to macroecological theory and to emerging patterns in the literature on trees, for example https://doi.org/10.1073/pnas.2003873117 (see Fig. 2), as well as other papers already cited in the manuscript.

Response: We appreciate this suggestion and agree that examining the diversity of life-history strategies across environmental gradients is an important direction for future research. While this was beyond the scope of the present study, we now briefly acknowledge this perspective in the Discussion (from L337).

Finally, I would be a bit more cautious in discussing the relationship between temperature and growth in ways that imply how species will respond to rapid climate change. The present results seem more naturally interpreted as evidence of broad biogeographic patterns in mean growth and in the diversity of growth strategies under current climate conditions. These patterns are likely shaped by long-term evolutionary processes, local adaptation, and abiotic filtering, rather than directly demonstrating shifts in species-level demography due to climate change. This is a relatively minor point, but I think a slightly more restrained interpretation would improve the framing.

Response: We thank the reviewer for this important point and agree that our results are more appropriately interpreted as reflecting broad biogeographic patterns under current climate conditions, shaped by long-term evolutionary processes, local adaptation, and abiotic filtering.

We have revised the Discussion accordingly (from L286) to adopt this interpretation and to avoid direct inference about species-level demographic responses to ongoing climate change. At the same time, we retain discussion of potential implications, but now frame these more cautiously, focusing on possible shifts in species composition and associated changes in plant longevity.

We have also made corresponding adjustments in the Abstract and Introduction to ensure consistent and more restrained framing throughout the manuscript.

I did not fully understand the interpretation of "mediated by temperature" (around line 168). If the goal is to estimate a mediated effect, why not use a structural equation model, as Fig. 4 seems to imply visually? I may simply be missing something, but this point would benefit from clearer explanation.

Response: We thank the reviewer for this valuable and constructive suggestion. In the original analysis, we assessed potential mediation of the temperature–longevity relationship by including growth as a covariate in the longevity–temperature model. This approach allowed us to test whether the association between temperature and longevity persisted after accounting for variation in growth, thereby providing an indirect assessment of mediation.

Following the reviewer's suggestion, we have now implemented a structural equation modelling (SEM) framework to explicitly disentangle direct and indirect pathways linking mean annual temperature (MAT), growth, and longevity. This approach enables a formal test of whether the effect of temperature on longevity is mediated through growth.

The SEM results are fully consistent with our original analysis, indicating that temperature influences longevity both directly and indirectly via its effect on growth. These results strengthen our interpretation rather than alter it.

We now present the SEM analysis in the Results section (from L192), in Supplementary Table 2, and in a revised Figure 4, which includes a path diagram with standardized coefficients. In addition, we have revised the corresponding text to clarify the distinction between the initial regression-based approach and the SEM framework, and to explicitly explain the concept of mediation used in this study.

Comment on the Abstract:"Pace of life" is a broad term that captures the timing of events across the life cycle, including growth strategies, age and size at maturity, and life expectancy, rather than referring specifically to life expectancy. I would suggest a small edit here for clarity.

Response: We thank the reviewer for this suggestion and have revised the Abstract accordingly to improve clarity.

We hope that the revisions and clarifications provided in response to the reviewers' comments have improved the clarity, robustness, and overall framing of the manuscript. We believe that the revised version addresses all concerns raised and has been substantially strengthened as a result.

We thank you and the reviewers again for your time and constructive feedback, and we look forward to your further consideration of our manuscript.

Sincerely,
Jan Binter
on behalf of all co-authors
